# Factors associated with depressive symptoms in patients with benign prostatic enlargement

**DOI:** 10.4102/phcfm.v15i1.3572

**Published:** 2023-05-22

**Authors:** Husni H. Abdalla, Jasmit Shah, Tabitha A. N. Nyanja, Jacob S. Shabani

**Affiliations:** 1 Department of Family Medicine, Aga Khan University, Nairobi, Kenya; 2 Department of Internal Medicine, Aga Khan University, Nairobi, Kenya

**Keywords:** depression, depressive symptoms, benign prostatic enlargement (BPE), urology, family medicine, lower urinary tract

## Abstract

**Background:**

Depression is a common condition that may lead to suicide at its worst. It is considered one of the primary causes of morbidity globally. Among the urological causes of depression is benign prostatic enlargement (BPE).

**Aim:**

To determine the prevalence and factors associated with depressive symptoms among BPE patients.

**Setting:**

This study was conducted in the Urology and Family Medicine Clinic at the Aga Khan University Hospital, Nairobi and Urology clinic at the Aga Khan Hospital Mombasa.

**Methods:**

The study was a cross-sectional design recruiting 308 males above the age of 40. Patient Health Questionnaire-9 and International Prostate Symptom Score (IPSS) were used to assess depressive symptoms and lower urinary tract symptoms (LUTS), respectively. Association between depressive symptoms and LUTS was determined. Factors associated with depressive symptoms were analysed by logistic regression.

**Results:**

Prevalence of depressive symptoms among patients with symptomatic benign prostatic enlargement (sBPE) was 42.90%. Factors associated with depressive symptoms included comorbid conditions, medication side effects, reduced libido, alcohol use, disturbed sleep at night and anxiety in regard to the prostate condition.

**Conclusion:**

There is a high prevalence of depressive symptoms among men with BPE. Assessment and early intervention for depressive symptoms among men with BPE should be initiated before clinical depression sets in.

**Contribution:**

The study has created a knowledge base on factors associated with depressive symptoms among men with sBPE in the African context.

## Introduction

Depression is a widespread mental disorder, presenting with a constant feeling of low mood, hopelessness and lacking interest in pursuits once enjoyed over a period of at least 2 weeks, in addition to other diagnostic criteria.^1^ Depression at its worst may lead to suicide.^2^ The World Health Organization (WHO) states that depression is the primary cause of morbidity globally.^3^ The negative outcome of depression, in addition to suicide, includes loss of workplace productivity that can affect the economy, earlier mortality that is unrelated to suicide, poor adherence to medication and worse outcomes of comorbid medical conditions. Globally, more than 300 million individuals are experiencing depression, resulting in an estimated global prevalence of 4.4%.^4^ Kenya is ranked 5th highest in Africa in terms of depression prevalence with 1.9 million cases in a population of approximately 52 million people.^4^ There exists a complex bidirectional relationship between depression and lower urinary tract symptoms (LUTS) secondary to benign prostatic enlargement (BPE).^2^

Benign prostatic enlargement arises from benign prostatic hyperplasia (BPH).^5,6^ While BPH in itself is usually asymptomatic and does not require treatment, it may result in BPE. ^7^ Benign prostatic enlargement can obstruct the bladder and cause LUTS resulting in symptomatic benign prostatic enlargement (sBPE). Symptomatic benign prostatic enlargement is associated with a decreased quality of life (QoL) and can interfere with daily activities and function because of the LUTS experienced.^2^ Studies note that sBPE is associated with erectile dysfunction, sleep deprivation, loss of libido, impairment of daily functions and other related issues that are associated with depression.^8,9,10^

Lower urinary tract symptoms as a result of BPE often require clinical intervention for management.^7^ The occurrence of LUTS among individuals affected with BPE ranges from 50% to 80% depending on age.^11^

Some subsets of LUTS such as nocturia cause disturbed sleep, which may also lead to depression or increase its severity.^12^ In cases where patients had pre-existing depression, their subjective assessment tends to increase the LUTS severity, because of catastrophic cognitive thinking associated with the depression.^2^ As such, while LUTS may instigate depression, having depression likely worsens the experience of LUTS, which then exacerbates depressive symptoms, hence resulting in a vicious cycle. Other causes of depression in patients with BPE include comorbid conditions and impotence.^13^ Previous studies have revealed the prevalence of depressive symptoms in BPE as a variable ranging from 4.92%, 17.7% and 22.4%, in South Korea, China and Poland, respectively.^13,14,15^

There is a paucity of studies that have focused on sBPE and its interplay with depression in the African context and more specifically in Kenya.^16^ This is especially important given the unique socio-cultural, socio-economic and health system factors experienced within the African and Kenyan context. Social and cultural environments have a demonstrable link with the prognosis of various diseases.^17^ This article therefore sought to determine the prevalence and establish factors associated with depressive symptoms among patients diagnosed with sBPE.

## Research methods and design

### Study design

The study employed a cross-sectional descriptive design.

### Setting

The study was carried out in the Urology and Family Medicine Clinic (FMC) at the Aga Khan University Hospital (AKUH), Nairobi as well as the Urology Clinic in Aga Khan Hospital Mombasa (AKHM). Aga Khan University Hospital is one of the referral centres in East and Central Africa. While AKHM, a sister hospital is a referral centre along the Kenyan Coast.

The Urology clinic in AKUH is open 5 days a week during weekdays, while the Urology Clinic in AKHM is open 3 days a week: Mondays, Wednesdays and Fridays. The urologists in these clinics see a variety of urological conditions including BPE. The FMC in AKUH is open 6 days a week, from Mondays to Saturdays seeing a wide spectrum of conditions affecting the family including BPE. Patients are managed by family physicians in this clinic.

### Study population

Study participants comprised of male individuals aged 40 years and older attending the Urology and FMC.

### Inclusion criteria

The study participants included individuals diagnosed with sBPE based on medical history and physical assessment including digital rectal exam (DRE), and/or radiological examination, which was either trans-abdominal or trans-rectal ultrasound suggesting a prostate volume of ≥ 30 cm³ and/or prostate specific antigen (PSA) levels above 1.5 ng/ml.

### Exclusion criteria

The study excluded individuals with a diagnosis of cancer of the prostate (either clinical or histological report), prior history of mood disorder or currently on treatment for psychotic disorder, dementia, patients with an active urinary tract infection, patients with urethral stricture and patients with prostatitis.

### Sample size estimation

Sample size estimation was based on prevalence formula and literature.^15^ The minimum size of sample required for the study was 268 at 95% confidence level, prevalence of depression in BPH of 22.4% and 80% power. When an attrition rate of 15% was added, the minimum sample size derived was 308.

### Sampling procedure

Convenient sampling was used based on the eligibility criteria. A trained research assistant identified men with sBPE as per study protocol in the above clinics at the two hospitals. The research study was explained to triaged clients who met the study requirements. Due to the coronavirus disease 2019 (COVID-19) pandemic, there was a low turnout of participants in the clinics. As such, approval was sought from the hospital ethics committee to recruit participants who had previously been diagnosed with BPE either in the urology clinics or FMC and had met the study criteria. The patients’ information was sought from the medical records department in both AKUHN and AKHM (who had previously been seen in the above clinics), in addition to those recruited in the clinics. Qualified participants were contacted via telephone call and informed about the study. Patients who agreed to participate in the study were taken through the consent form and verbal consent was obtained. An appointment for the interview was then arranged at the clients’ convenience.

### Data collection

To categorise patient characteristics, depressive symptoms and the severity of LUTS better, a structured questionnaire divided into three parts was utilised to collect data:

- Information on socio-demographic data, anthropometric measurements, lifestyle and medical history were gathered.
- Questions on depressive symptoms were assessed using the Patient Health Questionnaire-9 (PHQ-9), which was instrumental in capturing empirical scores for depression.^18^ The PHQ-9 has been used previously in a Kenyan population, which is the population of interest in this study.^19^ Each response to the nine questions was scored between zero and three on a Likert scale and the final score ranged from 0 to 27. The higher the score, the more severe the depressive symptoms. Anyone who scored 5 and above in PHQ-9 was considered to have depressive symptoms.
- The severity of LUTS was assessed using the International Prostate Symptom Score (IPSS).^20^ Each response to the seven questions was scored between zero and five on a Likert scale and the final score ranged from 0 to 35. The higher the score, the more severe the LUTS.

### Quality control

The above research tools as well as the consent form were translated into the Kiswahili language in order to include Swahili speakers in the study, which is the national language of Kenya. A language teacher who was conversant with both English and Kiswahili had gone through the Kiswahili version. He was able to back translate the above tools into English without losing the original meaning.

To verify the workability of the study tools, the questionnaire was piloted among men with BPE in a clinic attended by patients with similar features to those recruited in the study. This was done prior to collecting data from study participants. The pilot data were analysed to determine the internal reliability of the PHQ-9 and IPSS. The pilot results showed that the PHQ-9 had a Cronbach’s alpha of 0.80, while the IPSS had a Cronbach’s alpha of 0.85.

Collected data were physically verified by the principal investigator for accuracy. These were then recorded in REDCap (Research Electronic Data Capture), a data management tool used in this study.^21^ The data were then transferred to Statistical Package for Social Sciences (SPSS) version 25, for analysis.

### Data analysis

Descriptive statistics were summarised as frequencies and percentages for categorical variables, whereas continuous data were summarised as medians and interquartile range. The association between depression and other variables was tested using Fisher’s exact test. The association of these factors with depressive symptoms was illustrated using odds ratio (OR). Multivariate analysis using logistic regression was utilised in the assessment of the associations between depressive symptoms and the independent variable (LUTS). This was adjusted for confounders (like age, alcohol use, education, sleep problems and fear of prostate problems) that were found in the univariate analyses with a *p*-value of less than 0.1. Adjusted odds ratio (AOR) showed the strength of association between the severity of LUTS and depression.

### Ethical considerations

Ethical clearance to conduct this study was obtained from the Aga Khan University, Nairobi Institutional Ethics Committee, reference number 2019/IERC-58, and research permit obtained from the National Commission for Science, Technology and Innovation (NACOSTI) reference number 622433.

## Results

An aggregate of 308 respondents were recruited in the study out of 340 participants approached. Of the 32 participants excluded, 11 had prostate cancer, four had dementia, two had major depressive disorder (MDD) before the diagnosis of BPE and 15 refused to participate in the study. The baseline demographic characteristics of the study participants are shown in Table 1.

**TABLE 1:** Baseline demographic characteristics of participants (*N*= 308).

Baseline demographic characteristics	*n*	%	Median	IQR
Age (years)	-	-	61.00	55.0, 69.0
Age groups				
≤ 50 years	54	17.53	-	-
51–60 years	90	29.22	-	-
> 60 years	164	53.25	-	-
Weight (Kg)	-	-	79.00	73.0, 82.0
Height (m)	-	-	1.69	1.68, 1.70
**BMI Categories**				
Normal †	4	1.30	-	-
Underweight‡	43	14.00	-	-
Overweight §	203	65.90	-	-
Obese ¶	58	18.80	-	-
**Marital status**				
Single	1	0.30	-	-
Married	288	93.80	-	-
Divorced or Separated	4	1.30	-	-
Widowed	14	4.60	-	-
**Education level**				
Primary	11	3.60	-	-
Secondary	68	22.10	-	-
College Diploma	84	27.30	-	-
University	145	47.10	-	-
**IPSS LUTS Severity Categories**				
Mild (1–7)	98	31.80	-	-
Moderate (8–19)	193	62.70	-	-
Severe (20–35)	17	5.50	-	-
**Sexually active**				
Yes	259	87.20	-	-
No	38	12.80	-	-
**Problems from sexual activeness**				
Yes	197	76.10	-	-
No	62	23.90	-	-
**Problems experienced**				
Erectile dysfunction	112	56.90	-	-
Ejaculation problems	31	15.70	-	-
Reduced libido (sexual drive)	117	59.40	-	-
Other – painful erection	1	0.50	-	-
**Smoking**				
Yes	49	15.90	-	-
No	259	84.10	-	-
**Alcohol use**				
Yes	96	31.20	-	-
No	212	68.80	-	-

IQR, Interquartile range; Kg, Kilogram; m, metres; %, percentage; IPSS, International Prostate Symptom Score; LUTS, lower urinary tract symptoms; BMI, body mass index.

† , 18.5kg/m²–24.9kg/m²;

‡ , < 18.5kg/m²;

§ , 25 kg/m²–29.9 kg/m²;

¶ , ≥ 30 kg/m².

The study had more participants aged above 60 years (*n* = 164, 53.25%), those who were overweight (*n* = 203, 65.90%), married (*n* = 288, 93.80%) and individuals with university degrees (*n* = 145, 47.10%). The median age of the respondents was 61 years, while the age ranged from 40 years to 81 years. One-fifth of the study participants were obese. This study had 62.70% of patients with moderate LUTS and 5.50% of patients with severe LUTS accounting for over two-thirds (68.2%) of the study population.

### Prevalence of depressive symptoms among benign prostatic enlargement patients

The severity of depression was categorised according to the PHQ-9 cut-off points as shown in Table 2. Out of the study population of 308, 39.90% had mild depression (5–9), 2.30% had moderate depression (10–14) and 0.30% had moderately severe (15–19) or severe depression (20–27). The majority of those depressed had mild symptoms.

**TABLE 2:** Depression categories (*N* = 308).

Depressive symptoms level	*n*	%	95% CI
None †	176	57.10	51.41%, 62.74%
Mild ‡	123	39.90	34.42%, 45.64%
Moderate §	7	2.30	0.92%, 4.63%
Moderately severe ¶	1	0.30	0.0%, 1.80%
Severe ††	1	0.30	0.0%, 1.80%
Depressive symptoms ≥ 5	132	42.80	-
No depressive symptoms < 5	176	57.10	-

† , 0–4;

‡ , 5–9;

§ , 10–14;

¶ , 15–19;

†† , 20–27.

CI, lower and upper confidence limit for the 95% confidence interval of odds ratio.

Note: *P*-value < 0.05.

The results revealed that slightly above two-fifths (42.8%) of the respondents had depressive symptoms as shown in Table 2. Those who scored 5 and above from the PHQ-9 constituted 132 individuals out of 308 participants. Individuals with MDD constituted 2.90%, using cut-off score of at least 10 or above using the PHQ-9 questionnaire.

### Factors associated with depressive symptoms in patients diagnosed with symptomatic benign prostatic enlargement

The results in Table 3 revealed that patients with BPE who had co-morbidities, those who reported medication side effects, those experiencing sexual problems, those consuming alcohol, those who experienced problems during sleep at night and those with anxiety surrounding their prostate condition had a higher likelihood of having depression compared to those who were not. Among those with sexual problems, only those with reduced libido had higher chances of getting depressed.

**TABLE 3:** Factors associated with depressive symptoms in patients diagnosed with symptomatic benign prostatic enlargement (*N =* 308).

Variable	OR	95 % CI	*p-* value
**BMI**			
Normal †		1 Reference	
Underweight ‡	0.563	0.054–5.875	0.631
Overweight §	1.216	0.617–2.395	0.573
Obese ¶	1.808	0.809–4.043	0.149
**Surgical procedure for prostate**			
No		1 Reference	
Yes	1.499	0.952–2.361	0.081
**Co-morbidities**			
No		1 Reference	
Yes	1.705	1.068–2.722	0.025
**Medication side effect**			
No		1 Reference	
Yes	2.833	1.775–4.523	< 0.001
**Experiencing sexual problem**			
No		1 Reference	
Yes	2.014	1.088–3.730	0.026
**Erectile dysfunction**			
No		1 Reference	
Yes	1.121	0.702–1.791	0.632
**Ejaculation problems**			
No		1 Reference	
Yes	0.959	0.452–2.034	0.913
**Reduced libido**			
No		1 Reference	
Yes	1.644	1.033–2.619	0.036
**Currently smoking**			
No		1 Reference	
Yes	3.294	0.277–39.138	0.345
**Alcohol use**			
No		1 Reference	
Yes	1.95	1.197–3.177	0.007
**Exercise regularly**			
No		1 Reference	
Yes	0.796	0.498–1.271	0.339
**Problems with sleep**			
No		1 Reference	
Yes	3.185	1.933–5.247	< 0.001
**Anxiety because of prostate condition**			
No		1 Reference	
Yes	4.103	1.367–12.308	0.012
**Level of education**			
Primary		1 Reference	
Secondary	7.436	0.901–61.398	0.063
College Diploma	8.667	1.061–70.765	0.044
University	7.683	0.958–61.602	0.055

BMI, body mass index; OR, odds ratio.

† , 18.5–24.9;

‡ , < 18.5;

§ , 25–29.9;

¶ , ≥ 30.

Note: Association between factors and depression, *p*-value < 0.05.

OR, odds ratio; 95 % CI, lower and upper confidence limit for the 95% confidence interval of odds ratio.

Among those with sexual dysfunction, individuals experiencing erectile dysfunction and ejaculation problems did not have a higher likelihood of getting depressed.

## Discussion

The outcome of the research revealed that 42.90% of the respondents had depressive symptoms, which is notably greater compared to previous studies. For instance, the prevalence of depressive symptoms in participants with BPE and/or LUTS in South Korea was 4.92%; in China, it was 17.7%, whereas in Poland, it was 22.4% (13–15). This study had 62.70% of patients with moderate LUTS and 5.50% of patients with severe LUTS accounting for over two-thirds (68.2%) of the study population. This may explain the increased proportion of patients experiencing depression in this study compared to other previous studies. While the prevalence of MDD was found to be 2.90%, which was less than the global prevalence estimate of depression (4.4%) and prevalence of depression in Kenya.^4^

In this study, erectile dysfunction did not have a significant association with depressive symptoms, even though the odds of one having depression increased if one experienced sexual dysfunction as a result of reduced libido. However, in a Polish study, depression was associated with erectile dysfunction as well as LUTS severity, nocturia, BPH treatment, sedentary lifestyle and co-morbidities.^15^ Sexual dysfunction reduces self-esteem and adversely affects relationships.^13^ Reduced libido prominently featured as a significant factor in increasing the chance of being depressed. Equally, erectile dysfunction and ejaculation problem is expected to be a significant factor, but in this study, patients with erectile dysfunction and ejaculation problems did not have a higher likelihood of being depressed. However, this unexpected observation could be explained by the stigma associated with sexual dysfunction where men do not talk openly about their sexuality. In this study, sleep disturbance at night mainly because of nocturia increased the odds of having depression, whereas a sedentary lifestyle was not found to be associated with depression.

The presence of co-morbid conditions in patients with BPE approximately doubled the chances of having depression in the study. This correlates with the results of another study that showed that the presence of co-morbidities in patients with BPE is associated with deterioration in the quality of life and depression.^9^ If these co-morbidities are not well managed, these conditions can deteriorate and contribute to the exacerbation of depressive symptoms among these patients.^9^

Medication side effects associated with BPH treatment increased the chances of having depression by almost three times in the present study. A study in Ontario showed similar results as revealed by an increase in the level of depression in the first 18 months of treatment with 5-alpha reductase inhibitors (5-ARI).^22^ A meta-analysis of men on 5-ARIs, however, differed with these findings and did not show an association between the medication and future risk of depression.^23^ The meta-analysis, however, revealed a marginal statistical significance with its inclination towards depression. Hence, patients on medical treatment for BPH should be closely monitored for side effects.

This study showed that individuals with BPE who experienced problems during sleep had higher chances of having depression threefold. Lower urinary tract symptoms especially nocturia are significantly associated with the development of sleep disturbances that cause deterioration of the patient’s overall QoL and increases depression risk.^8,24^ Providing optimal treatment of LUTS including nocturia secondary to BPE and other co-morbidities should reduce disturbed sleep at night, hence improving the quality of sleep and ultimately depressive symptoms.

The likelihood of BPH patients who consumed alcohol in the present study had double the risk of having depression as compared to those who did not drink. According to Boden et al, the existence of any of the two disorders doubles the risk of the other disorder, with AOR between 2.00–2.09.^25^ There is also a causal relationship between alcohol use disorder (AUD) and depression as one in which AUD increased the chances of depression.^25^ Patients with BPH who consume alcohol should be encouraged and counselled to quit drinking alcohol.

In this study, patients with anxiety about their prostate problems increased the chance of being depressed fourfold. A study of patients with BPE on 5-ARI revealed that the patients feared a reduction in the level of sexual function, which was associated with a reduced quality of life and increased risk of depression.^26^ A common fear among patients who had undergone transurethral resection of the prostate (TURP) or who were on 5-ARI was the loss of libido that significantly increases the risk of depressive symptoms.^23^ In another Korean study, BPE affected the daily function of patients especially as a result of urinary urgency that affected their quality of life.^26^ Clinicians should provide health education to patients in regard to the anticipated side effects of medication to allay patient fears.

### Limitations

This study was carried out in private hospitals in Kenya. The study sites mostly cater for patients from a higher socioeconomic status. The results therefore may not be representative of the general population. Convenient sampling was done because of pragmatic reasons as a result of time constraints and limited financial resources. Data were collected throughout the day from the patients who presented to the clinics as walk-in clients. This may have resulted in missing out on all eligible patients from the study population catchment area. However, the bias potentially brought about by convenient sampling is mitigated by the non-probable manner in which walk-in patients came to the clinics. Due to the COVID-19 pandemic, the number of patients seen in the Urology and FMC had reduced. The study sample consisted of patients who are both currently seen at the clinics as well as those previously seen in the above clinics. Those patients who presented to the clinics despite the pandemic could have been symptomatically different from those who did not come. The reasons for those who refused to participate in the study could be related to the LUTS or depression symptoms. These issues created a sampling bias. Different studies and clinicians diagnosed BPE differently. Previous studies had no universally agreed definition of BPE; hence it may be difficult to compare results across the populations. The diagnosis of depression was based solely on a score of PHQ-9. As a result time constraint and limited budgetary allocation, participants were not clinically assessed and diagnosed with a depressive episode.

Despite these limitations, this study has contributed to expanding the knowledge base about the factors associated with depressive symptoms among men diagnosed with BPE in the African context. This study is, therefore, instrumental in addressing a knowledge gap by highlighting the disease burden of patients affected with symptomatic BPE in relation to depression. Additionally, a Swahili-translated version of the tool was adopted in this study. This ensured non-English speakers were not eliminated from the study based on their limitation to communicate in English. This ensured the findings are all inclusive and can be generalised to a larger population.

### Recommendations

In regard to the study results, the authors propose prioritisation of the following recommendations regarding the mental health of men presenting with LUTS secondary to sBPE. Screening for depression among men with sBPE who are at risk of depression to ensure early detection and intervention. In the long term, the cost of managing patients with co-morbid BPE and depression may be less. This may simultaneously improve the patient’s quality of life and activities of daily living. Patients with associated factors for depression as observed in the study should be optimally treated to reduce the chance of progressing into clinical depression. The authors recommend a different study such as a prospective study or a population-based study be conducted on a larger and more representative population. This may allow for generalisability. The results may allow proper healthcare planning and prevention of the progression of mild depressive symptoms to MDD resulting in a reduction of medical costs if treatment for depression is initiated at an earlier stage.

## Conclusion

High prevalence of depressive symptoms was found among men with symptomatic BPE. Assessment and intervention of depressive symptoms in men with BPE should be initiated early before clinical depression sets in. This is because depression is associated with higher morbidity and mortality.

## References

[1] American Psychiatric Association. Diagnostic and statistical manual of mental disorders. 5th ed.; Washington: American Psychiatric Association; 2013.

[2] Dunphy C, Laor L, Te A, Kaplan S, Chughtai B. Relationship between depression and lower urinary tract symptoms secondary to benign prostatic hyperplasia. Rev Urol. 2015;17(2):51. 27222640 10.3909/riu0658 PMC4857895

[3] World Health Organisation. Depression. Geneva: World Health Organization. 2018.

[4] World Health Organisation. Depression and other common mental disorders: Global health estimates. Geneva: World Health Organization; 2017. Contract No.: WHO/MSD/MER/2017.2.

[5] Hecht SL, Hedges JC. Diagnostic work-up of lower urinary tract symptoms. Urol Clin. 2016;43(3):299–309. 10.1016/j.ucl.2016.04.002 27476123

[6] Roehrborn CG. Benign prostatic hyperplasia: an overview. Rev Urol. 2005;7(suppl 9):S3. PMC1477638 16985902

[7] McVary KT. Clinical manifestations and diagnostic evaluation of benign prostatic hyperplasia. Waltham, MA): Up To Date; 2019.

[8] Branche BL, Howard LE, Moreira DM, et al. Sleep problems are associated with development and progression of lower urinary tract symptoms: Results from REDUCE. J Urol. 2018;199(2):536–542. 10.1016/j.juro.2017.08.108 28870861

[9] Calogero AE, Burgio G, Condorelli RA, Cannarella R, La Vignera S. Epidemiology and risk factors of lower urinary tract symptoms/benign prostatic hyperplasia and erectile dysfunction. Aging Male. 2019;22(1):12–19. 10.1080/13685538.2018.1434772 29392976

[10] Fan Y-H, Lin AT, Huang Y-H, Chen K-K. Health care-seeking behavior in benign prostatic hyperplasia patients. Urol Sci. 2017;28(3):169–173. 10.1016/j.urols.2016.12.003

[11] Egan KB. The epidemiology of benign prostatic hyperplasia associated with lower urinary tract symptoms: prevalence and incident rates. Urol Clin. 2016;43(3):289–297. 10.1016/j.ucl.2016.04.001 27476122

[12] Breyer BN, Shindel AW, Erickson BA, Blaschko SD, Steers WD, Rosen RC. The association of depression, anxiety and nocturia: a systematic review. J Urol. 2013;190(3):953–957. 10.1016/j.juro.2013.03.126 23680309 PMC4153377

[13] Jeong WS, Choi HY, Nam JW, et al. Men with severe lower urinary tract symptoms are at increased risk of depression. Int Neurourol J. 2015;19(4):286. 10.5213/inj.2015.19.4.286 26739184 PMC4703937

[14] Choi EP, Lam CL, Chin WY. Mental health of Chinese primary care patients with lower urinary tract symptoms. Psychol Health Med. 2016;21(1):113–127. 10.1080/13548506.2015.1032309 25887131

[15] Pietrzyk B, Olszanecka-Glinianowicz M, Owczarek A, et al. Depressive symptoms in patients diagnosed with benign prostatic hyperplasia. Int Urol Nephrol. 2015;47(3):431–440. 10.1007/s11255-015-0920-5 25673555 PMC4341023

[16] Matoke VO. Health seeking behavior associated with prostatism among men aged over forty years in nyamira county, Kenya: Kenyatta University; 2018.

[17] Hernandez LM, Blazer DG. The impact of social and cultural environment on health. Genes, behavior, and the social environment: Moving beyond the nature/nurture debate, Washington: National Academies Press (US); 2006. 20669442

[18] Levis B, Benedetti A, Thombs BD. DEPRESsion Screening Data (DEPRESSD) Collaboration. Accuracy of Patient Health Questionnaire-9 (PHQ-9) for screening to detect major depression: individual participant data meta-analysis. 2019;365:1476. 10.1136/bmj.l1476 PMC6454318 30967483

[19] Monahan PO, Shacham E, Reece M, et al. Validity/reliability of PHQ-9 and PHQ-2 depression scales among adults living with HIV/AIDS in western Kenya. J Gen Intern Med. 2009;24(2):189. 10.1007/s11606-008-0846-z 19031037 PMC2629000

[20] Barry MJ, Fowler FJ, O’Leary MP, et al. The American Urological Association symptom index for benign prostatic hyperplasia. J Urol. 1992;148(5, Part 1): 1549–1557. 10.1016/S0022-5347(17)36966-5 1279218

[21] Harris PA, Taylor R, Minor BL, et al. The REDCap consortium: Building an international community of software platform partners. J Biomed Inform. 2019;95:103208. 10.1016/j.jbi.2019.103208 31078660 PMC7254481

[22] Welk B, McArthur E, Ordon M, Anderson KK, Hayward J, Dixon S. Association of suicidality and depression with 5α-reductase inhibitors. JAMA Intern Med. 2017;177(5):683–691. 10.1001/jamainternmed.2017.0089 28319231 PMC5818776

[23] Kim JH, Shim SR, Khandwala Y, Del Giudice F, Sorensen S, Chung BI. Risk of depression after 5 alpha reductase inhibitor medication: meta-analysis. World J Men’s Health. 2019;38(4):535–544. 10.5534/wjmh.190046 31190484 PMC7502319

[24] Baas WR, Butcher MJ, Lwin A, et al. A review of the FAERS data on 5-alpha reductase inhibitors: Implications for postfinasteride syndrome. Urology. 2018;120:143–149. 10.1016/j.urology.2018.06.022 29960004

[25] Boden JM, Fergusson DM. Alcohol and depression. Addiction. 2011;106(5):906–914. 10.1111/j.1360-0443.2010.03351.x 21382111

[26] Lee YI, Kim JW, Bae SR, et al. Effect of urgency symptoms on the risk of depression in community-dwelling elderly men. Korean J Urol. 2013;54(11):762–766. 10.4111/kju.2013.54.11.762 24255758 PMC3830969

